# 

**Published:** 1957-07

**Authors:** 


### *Contents*

| | | Page |
|---|---|---|
| A TOUR OF THE SOUTH-WESTERN PACIFIC | *Mr. A. L. Eyre-Brook* | 75 |
| THE PREVENTION OF ACUTE RHEUMATISM | *Prof. C. Bruce Perry* | 86 |
| HEAD INJURIES . . . . . | *Mr. G. L. Alexander* | 91 |
| BRISTOL AND BORDEAUX . . . . | *Dr. J. A. Cosh* | 98 |
| SARCOIDOSIS TREATED WITH CORTISONE | *Clinical Pathological Conference* | 101 |
| NOTES AND NEWS . . . . . . . | | 106 |
| APPOINTMENTS . . . . . . . . | | 107 |

